# Recent Electrochemical Advancements for Liquid-Biopsy Nucleic Acid Detection for Point-of-Care Prostate Cancer Diagnostics and Prognostics

**DOI:** 10.3390/bios14090443

**Published:** 2024-09-14

**Authors:** Joseph Broomfield, Melpomeni Kalofonou, Charlotte L. Bevan, Pantelis Georgiou

**Affiliations:** 1 Centre for BioInspired Technology, Department of Electrical and Electronic Engineering, Imperial College London, London SW7 2AZ, UK; j.broomfield20@imperial.ac.uk (J.B.); m.kalofonou@imperial.ac.uk (M.K.); 2 Imperial Centre for Translational and Experimental Medicine, Department of Surgery and Cancer, Imperial College London, London W12 0NN, UK

**Keywords:** point of care, prostate cancer, electrochemical devices, nucleic acids, EIS, voltammetry, chronoamperometry, field-effect transistors, ISFET

## Abstract

Current diagnostic and prognostic tests for prostate cancer require specialised laboratories and have low specificity for prostate cancer detection. As such, recent advancements in electrochemical devices for point of care (PoC) prostate cancer detection have seen significant interest. Liquid-biopsy detection of relevant circulating and exosomal nucleic acid markers presents the potential for minimally invasive testing. In combination, electrochemical devices and circulating DNA and RNA detection present an innovative approach for novel prostate cancer diagnostics, potentially directly within the clinic. Recent research in electrochemical impedance spectroscopy, voltammetry, chronoamperometry and potentiometric sensing using field-effect transistors will be discussed. Evaluation of the PoC relevance of these techniques and their fulfilment of the WHO’s REASSURED criteria for medical diagnostics is described. Further areas for exploration within electrochemical PoC testing and progression to clinical implementation for prostate cancer are assessed.

## 1. Introduction

Prostate cancer (PCa) presents a significant morbidity, where PCa mortality is the most common male-cancer-related death in 52 countries worldwide [1]. Prostate-specific antigen (PSA) testing is currently utilised to determine which men require further and more invasive testing. Population screening of PSA for PCa is not currently implemented, on account of the low specificity of the test to PCa [2,3]. Overtreatment and invasive diagnostic testing of men without PCa can result from high PSA levels in the blood, due to benign conditions [4]. Contrastingly, some PCa patients do not present with high circulating PSA concentrations, despite having the underlying disease.

Molecular diagnostic and prognostic testing for PCa has resulted in improved personalised medicine approaches for PCa. Nucleic acids, including DNA and RNA, can provide information on PCa presence, the chance of metastasis and the likelihood of clinical progression [5,6,7,8,9]. The Progensa prostate cancer antigen 3 (PCA3) mRNA assay has previously illustrated that nucleic acid molecular diagnostics can elevate the specificity of testing for PCa [10]. Specifically, the Progensa PCA3 urine test was utilised for PCa patients who had previously had a negative biopsy, to determine if they would require repeat biopsies [10]. However, a thorough economic evaluation of the Progensa PCA3 assay determined that the increase of quality-adjusted life years was not sufficient to accommodate the increased cost within the National Health Service in the United Kingdom [11]. The cell cycle risk (CCR) score (Myriad Genetics), Decipher score (Veracyte) and the genomic prostate score (GPS, Oncotype DX) are all marketed as prognostic mRNA panel tests [7,12,13]. While these tests represent the clinical utility of molecular diagnostics for PCa, each require specialised off-site laboratories and expensive equipment. Point of care (PoC) devices instead present the potential to bring personalised PCa diagnostics and prognostics directly within healthcare spaces. This could result in rapid multipanel testing of relevant biomarkers directly in the clinic, utilising non-specialised personnel.

**Figure 1:**
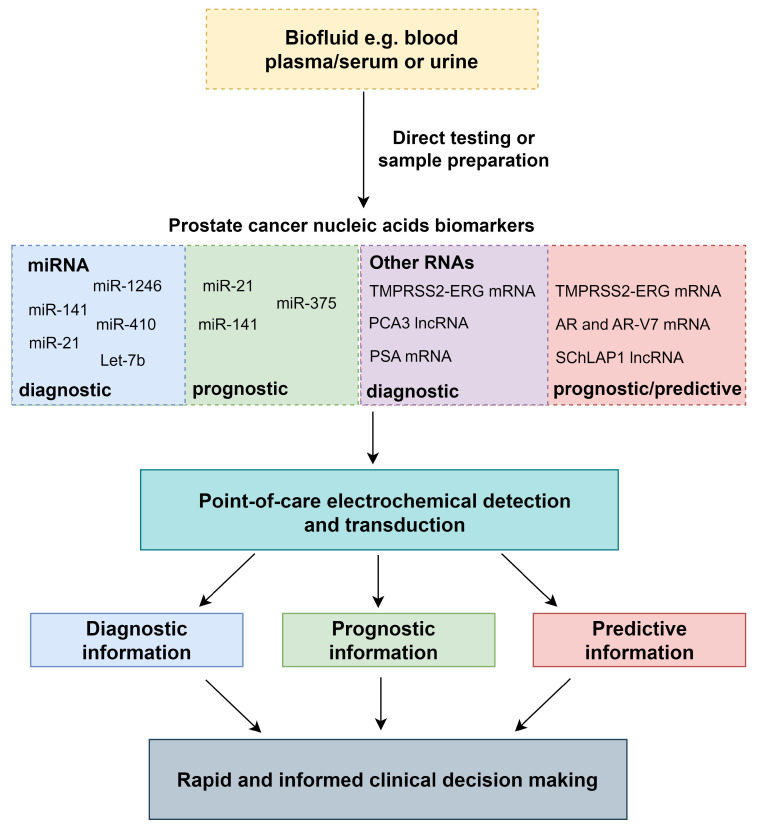
The generic process of nucleic acid detection from biofluids to clinical decision making with PoC electrochemical devices.

Electrochemical biosensors for nucleic acid detection can result in exceptionally high sensitivities rivalling the current gold standard for nucleic acid amplification tests (NAATs), the quantitative polymerase chain reaction (qPCR). However, qPCR requires specialised laboratories and bulky thermal cycling equipment [14]. Relevant circulating and exosomal nucleic acid markers previously detected with qPCR for PCa diagnosis and prognosis will be described in this work. Detection of these relevant nucleic acid markers with PoC-compatible methodologies for PCa have also been reported and will also be explored in this review. Electrochemical biosensors can provide relatively simple, inexpensive and rapid detection of target analytes, through biomarker recognition, signal transduction and electronic readout (Figure 1) [15]. Electrochemical impedance spectroscopy (EIS), voltammetry, chronoamperometry and potentiometric sensing using field-effect transistors (FETs) will be discussed as electrochemical techniques for nucleic acid PoC PCa testing.

The WHO has recommended that diagnostic PoC devices should adhere to the REASSURED criteria (Real-time connectivity, Ease of specimen collection, Affordable, Sensitive, Specific, User-friendly, Rapid and robust, Equipment-free or simple, and Deliverable to end users). Evaluation of current PoC tests for PCa will be discussed in relation to the REASSURED criteria, and further avenues for further research will be appraised.

## 2. Circulating and Exosomal Nucleic Acid Biomarkers for PCa Diagnosis and Prognosis

### 2.1. MicroRNAs

Detection of microRNAs—small (19–24 bp) single-stranded RNAs that predominantly regulate gene expression through the degradation of specific target mRNAs [16]—has seen significant interest in PCa for prospective diagnostics and prognostics. In blood and urine, microRNAs are inherently more stable than mRNAs [17,18]. As such, detection of microRNAs in both urine and blood could present easy, minimally invasive approaches to PoC nucleic acid detection. To date, several electrochemical PoC devices have targeted miR-21, miR-141, miR-410, miR-375, miR-1246 and Let7b for PCa diagnostics and prognostics [19,20,21,22,23].

In multiple cancers, including prostate, miR-21 and miR-141 are upregulated, and they are elevated in both the urine and blood of PCa patients relative to healthy men [24,25]. Correspondingly, they are both often utilised for PCa electrochemical diagnostic tests. Prognostically, miR-21 is associated with the epithelial-to-mesenchymal transition (EMT) in PCa, potentially linking miR-21 to more aggressive forms of the disease [26]. Meta-analysis of miR-21’s prognostic utility has indicated that the presence of miR-21 is strongly correlated with poor prognosis, a higher Gleason score and the prostate cancer stage [27]. When compared to healthy men, miR-141 is also upregulated in blood plasma and exosomes in PCa [24,28]. Metastatic PCa patients with elevated levels of miR-141 in serum also have more metastatic bone lesions, potentially highlighting the prognostic value of miR-141 detection [29].

It is also the case that miR-375 has been implicated in PCa and has previously been of interest for prognostic purposes. Higher levels of circulating miR-375 are present in more advanced disease, particularly within metastatic PCa, despite miR-375 having anti-invasive and anti-EMT properties [30,31,32,33]. Also, miR-375-related pathways are associated with taxane resistance in metastatic castration-resistant PCa mCRPC and differentiation to neuroendocrine PCa [31,34,35,36]. In combination with miR-141 detection, plasma miR-375 can also predict time to radiological progression [35]. As such, circulating miR-375 levels can provide useful prognostic and predictive information.

Furthermore, miR-410 has been successfully detected with PoC electrochemical devices for PCa [37], while miR-410-3p has previously been associated with the downregulation of the phosphatase and tensin homolog/protein kinase B/mammalian target of the rapamycin (PTEN/AKT/mTOR) pathway and is indicative of poorer PCa prognosis [38]. In PCa patients, miR-410-5p detection in blood serum was significantly higher when compared to healthy men [39]. As a result, miR-410 could provide relevant diagnostic information for PCa.

### 2.2. mRNAs and lncRNAs

While mRNAs and long non-coding RNAs (lncRNAs) are nucleic acid biomarkers prone to degradation, the development of the Progensa PCA3 assay has established that RNA detection can be relevant for early PCa diagnosis. Within this test, the relative quantity of PCA3 lncRNA is reported, relative to PSA mRNA concentration. Reported PCA3 score ratios above 25 can be utilised to improve the specificity of diagnostic testing when compared to PSA testing [10,40]. As such, several electrochemical devices aiming to improve upon current diagnostics utilise PCA3 lncRNA alone or in conjunction with PSA mRNA [41,42,43,44].

The gene fusion TMPRSS2-ERG has additionally been utilised as a predictive and prognostic marker for PCa. TMPRSS2-ERG is largely considered a PCa-specific biomarker and is indicative of an aggressive subset of the disease [45]. This fusion results in overexpression of the oncogene ERG driving progression towards metastasis [46,47]. In urine, TMPRSS2-ERG presence correlates with clinically significant PCa, tumour size and a high Gleason score at prostatectomy [48]. In the blood of mCRPC patients, several studies have recorded that patients with TMPRSS2-ERG mRNA presence respond poorly to taxane therapies, with reduced PSA progression-free survival and overall survival within this patient cohort [49,50]. TMPRSS2-ERG mRNA detection, therefore, can act as a biomarker for PCa prognosis or as a potential predictive marker for taxane resistance.

The androgen receptor (AR) signalling pathway, while maintaining a prostatic differentiated function in healthy men, can drive PCa growth and invasion [51]. Targeted therapies of the AR are utilised as first-line treatments for inoperable disease. Castration resistance in PCa results from resistance to these therapies and is indicative of late-stage and ultimately terminal disease [52]. Several mechanisms for castration resistance have been determined, including amplification of the AR or the presence of constitutively active AR variants (AR-Vs) [53,54]. Of note, AR-V7 is deficient in the ligand-binding domain region and, therefore, indicative of resistance to androgen-deprivation therapy [53,55]. Within the blood, AR mRNA amplification is associated with reduced overall survival (OS) and progression-free survival (PFS) in patients treated with androgen-deprivation therapies (ADT) [54]. On account of the low abundance of these mRNA biomarkers in the blood, ultrasensitive amplification strategies, including digital droplet PCR (ddPCR), have previously been utilised [56,57,58]. PoC devices, however, could provide valuable predictive and prognostic information with less complex detection methods.

### 2.3. Biofluid Considerations and Sample Preparation

Electrochemical PoC tests will either directly detect the nucleic acid markers from the desired biofluid or after sample preparation, once the biomarkers are extracted and purified. Blood, urine and exosomal nucleic acids can provide valuable information for PCa diagnostics and prognostics. Urine contains prostatic secretions and can be collected non-invasively, avoiding potentially painful procedures for use in diagnostic or prognostic tests [59]. The Progensa PCA3 assay test has previously used urine for PCa diagnostics [10]. For electrochemical devices that directly test from urine samples, robust analysis will be required, to account for the high variability of pH, metabolites and solid particulates between patients [59,60,61]. Ensuring that the sensitivity and signal transduction of these devices is maintained could reduce the likelihood of false negatives. For nucleic acid detection from urinary exosomes the gold standard is ultracentrifugation followed by purification. Alternative approaches will be required to avoid the use of the highly specialised, bulky laboratory equipment currently utilised for this technique [62].

Detection of nucleic acids in the blood can additionally be utilised for PCa diagnosis and prognosis [32,35,63]. Since intravasation of tumour cells from the primary tumour occurs during the progression to metastasis, the presence of nucleic acids within the blood can be utilised for prognostic purposes for PCa. Electrochemical detection of microRNAs has previously taken place directly from patient samples for PCa, despite their low abundance [20,64]. Other electrochemical techniques have additionally been shown to detect low levels of synthetic microRNAs spiked into serum or plasma [19,37,65]. Specialised kits for circulating RNA extraction from the blood can also be utilised that typically result in cell lysis, protein denaturation followed by RNA purification [66]. However, there can be significant variation in yield and purity between kits and extraction methodologies [67]; mRNAs are low-abundance and labile biomarkers in the blood. For example, AR-V7 mRNA has previously been recorded in the magnitude of 0–146 copies per mL of blood from mCRPC patients [57]. Therefore, direct detection of mRNA in blood plasma and serum is likely to require high volumes and highly sensitive detection methods to be viable. Fragmentation of the mRNA in the blood can additionally present challenges for detection [64]. However, on account of the limited time that mRNAs remain in the blood, they can provide valuable temporal information on the PCa tumour state. To take place successfully, mRNA detection in the blood is more likely to require sample preparation and purification. Incorporation of PoC sample preparation techniques for mRNA detection, therefore, would be essential to bringing PoC electrochemical devices directly into the clinic.

## 3. Point-of-Care Electrochemical Techniques

### 3.1. Electrochemical Impedance Spectroscopy

**Figure 2:**
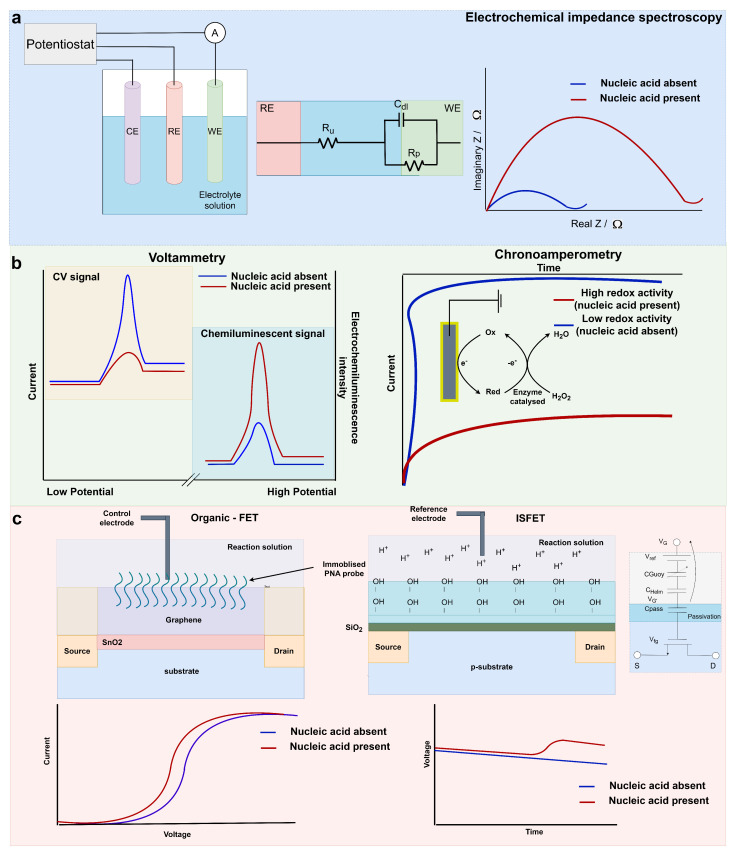
(**a**) A simplistic three-electrode electrochemical system and the graphical output of an EIS on−switch system like that reported in [81]; (**b**) An example cyclic voltammetry signal (off−switch) and electrochemiluminescence (on−switch), as reported by [82] for miR−21 and miR−141 detection. An example chronoamperometry output and redox system for nucleic acid biomarker detection similar to that reported in [83]; (**c**) Organic graphene FET cross section and graphical interpretation of the sensor output reported by [21] and ISFET cross section ISFET macromodel, and graphical interpretation of a sensor output for nucleic acid biomarker detection in [84].

EIS is a widely used point-of-care electrochemical technique for the diagnosis of cancers and infectious diseases [19,68]. In faradaic EIS, a redox solution (often [Fe(CN)_6_]^3−/4−^) is utilised. With a signal-on EIS biosensor, the target analyte presence at the working electrode reduces the flux of the electron exchange of the redox reaction (Figure 2a) [69]. The perturbation of the solution is then measured when an alternating current or voltage is applied across a range of frequencies. The steady-state nature of EIS can allow for high-sensitivity devices that can often be label-free [70]. Like many electrochemical techniques, appropriate functionalisation of the working electrode in EIS can result in the detection of proteins, metabolites and nucleic acids [71,72,73,74,75].

Detection of long non-coding RNA (lncRNA) PCA3 through a low-cost impedimetric sensor was reported by Coatrini Soares et al. PCA3 overexpression is a PCa-specific biomarker, and, therefore, it is utilised for potential early PCa diagnosis [76]. Single-stranded DNA (ssDNA) complementary to a region of the PCA3 lncRNA was immobilised on multi-walled carbon nanotube (MWCNT)-coated interdigitated gold electrodes. Binding of the PCA3 lncRNA resulted in impedance of the redox reaction present in the electrolyte solution. The specificity of the device was successfully confirmed with RNA extracted from PCa and HeLa cell lines. Quantitative detection of synthetic PCA3 RNA was achieved down to 0.128 nM [73]. The subsequent alteration of the working electrode to a printed carbon electrode coated with chondroitin sulfate stabilised gold nanoparticles (AuNPs) improved the sensitivity of PCA3 lncRNA detection to 83 pM [77]. This work presents a potential low-cost, sensitive and specific biosensor for PCA3 lncRNA detection at the PoC. Further exploration of quantitative PCA3 detection directly within clinical urine samples would illustrate the potential of this biosensor for early diagnosis of PCa.

Aptamers as recognition elements have additionally seen success in the detection of PCA3 lncRNA for EIS. Takita et al. developed a biosensor with PoC potential, using a screen-printed carbon electrode (SPCE) and AuNPs that could easily immobilise the aptamer with thiol chemistries [43]. Previous validation of the aptamer established a high affinity and specificity for PCA3 lncRNA binding [78]. Detection of PCA3 lncRNA down to 1 fM was observed with this method, and 20 fM could be detected in artificial serum samples [43]. Specificity testing with cell lines would additionally aid the biosensor for clinical implementation.

Since microRNAs are small single-stranded RNA sequences, complementary ssDNA probes can often be utilised as simple bio-recognition elements. Yaman et al. successfully and sensitively detected miR-410 with EIS and an AuNP assembled peptides nanotube graphite sensor [37]. Binding of miR-410 to a complementary ssDNA probe impeded the flux of a redox probe, and this steric hindrance could be quantitatively determined as an increase in resistance. Detection of miR-410 presence could be easily distinguished from miR-192 and miR-200c microRNAs. Recovery of spiked-in microRNA into human serum within this work illustrated the use of the device directly with biological mediums [37].

Despite the valuable utility of EIS for PoC nucleic acid detection, PCa testing directly from clinical samples for microRNA and mRNA biomarker detection has not yet taken place. However, previous impedimetric-based sensors have been successful in detecting PCa metabolites in patient samples, or nucleic acid markers in other cancers [79,80]. This illustrates the potential for clinical sample testing with EIS PoC devices for PCa.

### 3.2. Voltammetry and Chronoamperometry

#### 3.2.1. Voltammetry

Voltammetry is utilised to observe changes in current when the potential is adjusted at a fixed rate. The half-cell potential of a target analyte can be observed with voltammetric biosensors. Common voltammetry techniques for use within biosensors include cyclic voltammetry (CV), differential-pulse voltammetry (DPV) and square-wave voltammetry (SWV). Cyclic voltammetry can be utilised to observe the oxidation and reduction potentials of species in solution through forward and reverse sweeps of potential with a potentiostat [85]. In the switch-off CV example shown in Figure 2b, the nucleic acid presence quenches the electron exchange of ferrocene at the working electrode, reducing the amplitude of the current during the CV scans. Usage of CV can result in the formation of low-cost and simplistic biosensors for nucleic acid detection [82,86]. In DPV, a linear ramp with small pulses is utilised. The current is recorded before each individual pulse, reducing the effect of the charging current [87,88]. In SWV, the staircase potential is superimposed with a square wave, and the current is sampled after each pulse [89]. As a result, both techniques have low capacitive currents and high sensitivities for analyte detection.

A CV and chemiluminescence biosensor developed by Feng et al. was utilised for multiplexed detection of miR-21 and miR-141 (Figure 2b) [82]. This biosensor comprised dual working electrodes coated in AuNPs. A ruthenium luminophore complex was attached to the working electrode surface. Hairpin DNA probes for miR-21 and miR-141 detection were attached to ferrocene and immobilised on the working electrode surface. The ferrocene acted as a quencher to the ruthenium complex. Specific microRNA binding to the DNA hairpin probe quantitatively unquenched the luminophore. Since the proximity of the ferrocene to the working electrode was abrogated, CV negative readings were also indicative of microRNA binding [82]. Detection of miR-21 and miR-141 occurred down to concentrations of 6.3 fM and 8.6 fM, respectively. Robust detection of spiked samples into human serum samples illustrated the potential of the biosensor for testing directly with clinical samples.

Similarly, DPV was also utilised as an electrochemical detection technique for miR-21 presence as a diagnostic test for PCa [65]. Cd^2+^ ions, which intercalated with the miR-21 phosphate backbone, provided the signal for the DPV detection. Immobilisation of the peptide nucleic acid recognition element was conducted with a dendritic gold (den-Au) nanostructure attached to SWCNTs on a doped tin oxide electrode [65]. Den-Au nanostructures can greatly increase the surface area of the biosensor for probe immobilisation [90]. A limit of detection of 0.01 fM was observed with this method, and a 10 fM miR-21 presence spiked into serum samples was observed with good recovery (97%). However, since miR-21 is also upregulated in bladder cancer, detection in urine is likely to not be specific to PCa [24,91]. For a PoC device, multiplexing of miR-21 with other PCa-specific nucleic acids or as a companion to PSA testing could alleviate this issue.

Furthermore, miR-21 and miR-141 simultaneous detection with SVW has been successful, with potential point-of-care deployment and a dual working electrode system [92]. In this instance, the addition of duplex-specific nuclease-assisted target-recycling signal amplification improved the sensitivity of the device. This allowed for the rebinding of the original target to the probe, to boost the signal from the analyte presence [92]. Extracted RNA from 22Rv1 PCa and MCF-7 breast cancer cell lines were utilised to confirm the specificity of the device.

Signal amplification with SWV has additionally allowed for miR-21 and miR-141 detection down to 0.1 fM [19]. Tian et al. reported a paper-based electrode system utilising molybdenum sulphide AuNPs. Enhancement of the electrochemical device with platinum/copper metal–organic frameworks allowed for the high sensitivity of the device. The DNA probe for miR-21 was bound to ferrocene, and the miR-141 probe was bound to methylene blue. Distinction of these two biomarkers was, therefore, possible through the different oxidation/reduction potentials of their respective electrochemical reporters. High recoveries of both microRNAs were additionally recorded down to 20 pM within spiked serum samples [19].

A more simplistic SWV technique for miR-375 detection saw improved sensitivity down to 11.7 aM and a limit of detection of 12.4 aM in diluted serum [23]. In this work, an ssDNA probe complementary to miR-375 was immobilised onto a gold electrode with a standard thiol chemistry approach. With this method, miR-375 was only observed in metastatic PCa cell lines and not in immortalised prostate epithelial cell lines [23]. The high sensitivity and simplistic development of this SWV detection platform could introduce a facile, amplification-free method for PoC microRNA detection.

#### 3.2.2. Chronoamperometry

Chronoamperometry measures the current response over time during a single or double potential step. In the example shown in Figure 2b, the potential step induces a redox reaction involving 3,3′5,5′-tetramethylbenzidine (TMB) and horseradish peroxidase (HRP) when the target miRNA is present [83]. As a result, an observable shift in current over time is detected. Chronoamperometry has been utilised for PCa diagnostic purposes through the detection of PCA3 lncRNA and PSA mRNA [42]. Since the Progensa assay has previously established the clinical utility of detecting the ratio of PCA3 and PSA, emulation of this assay with PoC compatibility could result in a robust diagnostic test. Sanchez-Salcedo et al. utilised a sandwich assay for recognition of the two RNA biomarkers on individual working electrodes for multiplex detection. Binding of the target analytes resulted in the increased proximity of TMB to the working electrodes, to produce an electrochemical signal. The sensitivity of the device could detect PCA3 lncRNA and PSA mRNA down to 4.4 pM and 1.5 pM, respectively [42]. Importantly, detection of these biomarkers was additionally observed in PCa urine samples, where RNA was extracted and specifically enriched for PCA3 lncRNA and PSA mRNA. This work showed preliminary evidence of the clinical utility of the device. Further analysis with a larger cohort could determine the success of the device for early PCa diagnosis.

Similarly, chronoamperometry and reverse-transcription loop-mediated isothermal amplification (RT-LAMP) were utilised for PCA3 lncRNA and PSA mRNA detection [41]. LAMP is an isothermal amplification technique, often utilised for nucleic acid detection at PoC [93,94]. Here, LAMP was utilised to preamplify PCA3 lncRNA and PSA mRNA with a digoxigenin-bound dUTP. The LAMP amplicons were captured with strepavidin bound to an antidigoxigenin antibody and HRP. If the RNA target was present, accumulation of HRP occurred at the working electrode, and the reduction of benzoquinone to hydroquinone induced an electrochemical signal for chronoamperotery detection [41]. Extracted RNA from nine cell lines was utilised to robustly confirm the specificity of the electrochemical biosensor. Analysis with PCa and healthy urine samples established that PCA3 lncRNA and PCA3/PSA ratio were upregulated in PCa patients. All the steps required for this biosensor, except RNA extraction, can take place at the PoC. Evidence of quantitative RNA detection would further improve the value of PCA3/PSA ratios determined by this device.

An alternative chronoamperometry biosensor with an alternative isothermal reaction, recombinase polymerase amplification (RPA), was successful in detecting several nucleic acid targets, including TMPRSS2-ERG mRNA, PCA3 lncRNA, KLK2 mRNA (as an internal control) and SChLAP1 lncRNA. This work additionally presented a PoC methodology for sample preparation, utilising magnetic beads rendering extracted DNA and RNA sequences. RPA forward primers for each nucleic acid target were tethered to the working electrode, creating amplicons at the electrode surface in the presence of their respective targets. Peroxidase-mimicking nanozymes were subsequently added, which catalysed a redox reaction in the presence of the amplicons, rendering a detectable signal with chronoamperometry [95]. PCa cell lines (DuCaPs, LnCaPs and 22Rv1s) were utilised to confirm the specificity of the biosensor to the RNA targets. Simultaneous detection of the four nucleic acid targets in both the serum and the urine of PCa patients was achieved, and both biofluids were congruent for biomarker detection. Overexpression of these PCa biomarkers correlated with high-grade PCa [95]. This work illustrated a truly PoC method from sample to result, validated with a small cohort of PCa clinical samples within 30 min (Table 1).

**Table 1:** Summary of point-of-care bioelectrical nucleic acid devices for prostate cancer diagnosis and prognosis.

Bio-Electrical Detection Method	Bio-Recognition Element	Nucleic Acid Target	Limit of Detection	Quantitative Range	Endogenous Detection	References
EIS	ssDNA probe on chitosan and carbon nanotubes	PCA3 lncRNA	0.128 nM	N/A	cell line	[73]
EIS	printed carbon electrode, chondroitin sulfate stabilised AuNPs and ssDNA probe	PCA3 lncRNA	83 pM	N/A	N/A	[77]
EIS	SPCE, AuNPs and aptamer	PCA3 lncRNA	1 fM	0.1 pM to 10 nM	spiked artificial urine	[43]
EIS	AuNPs, peptide nanotubes and ssDNA probe	miR-410	3.9 fM	10 fM to 300 pM	spiked serum	[37]
chronoamperometry	framework nucleic acid electrode and ssDNA probe	miR-21, miR-141 and Let-7a	10 fM (miR-21) and 1 aM (miR-141)	10 aM to 1 pM (miR-141)	cell line	[96]
chronoamperometry	RPA and peroxidase-mimicking nanozymes	TMPRSS2-ERG, PCA3, SChLAP1 and KLK2 nucleic acids	50 copies	N/A	urine and serum samples	[95]
chronoamperometry	screen-printed carbon electrode and biotinylated ssDNA probe	exosomal miR-451 and miR-21	10 pM	10 pM to 100 nM	extracted exosomal RNA from urine samples	[83]
chronoamperometry	gold nanoparticles and sandwich assay	PCA3 and PSA mRNA	4.4 and 1.5 pM	25 pM to 10 nM (PCA3), 25 pM to 1 nM (PSA)	extracted RNA from urine samples	[42]
chronoamperometry	RT-LAMP, magnetic beads and SPCE	PCA3 lncRNA and PSA mRNA	N/A	N/A	extracted RNA from urine samples	[41]
chemoluminescence and CV	AuNPs, Ru complexes and DNA probes	miR-21 and miR-141	6.3 and 8.6 fM	0.02 pM to 150 pM (miR-21), 0.03 pM to 150 pM (miR-141)	N/A	[82]
DPV	SWCNT dendritic Au nanostructure and peptide nucleic acid probe	miR-21	0.01 fM	0.01 fM to 1 μM	spiked serum	[65]
SWV and EIS	MoS_2_/AuNPs/AgNW and signal amplification	miR-21 and miR-141	0.1 fM	1 fM to 1 nM	spiked serum	[19]
SWV	redox labelled DNA hairpins on Au electrode and recycling signal amplification	miR-21 and miR-141	4.2 and 3.0 fM	5 fM to 50 pM	cell lines	[92]
SWV	ssDNA probe and gold working electrode	miR-375	11.7 aM	10 aM to 1 nM	cell lines and spiked serum	[23]
graphene FET	peptide nucleic acids immobilised on graphene oxide nanosheet	miR-21, miR-1246 and Let-7b	10 fM	10 fM to 10 nM	urine samples	[21]
solution-gated graphene FET	ssDNA probe immobolised on Au gate	miR-21	0.01 aM	0.01 aM to 1 pM	blood serum patient samples	[20]
ISFET	target-specific RT-LAMP and pH-sensing passivation layer	AR-V7, TMPRSS2-ERG, YAP1 and AR-FL mRNA	5–8 aM	5–8 aM to 5–8 pM	cell lines, spiked serum and plasma	[84,97]

MicroRNA detection with chronoamperometry has additionally resulted in highly sensitive PoC electrochemical devices. In research conducted by Wen et al. [96], miR-21 and miR-141 and Let-7a detection occurred with a framework nucleic acid (FNA) platform. Formation of a tetrahedral nucleic acid framework interface on the surface of a gold working electrode resulted in microRNA binding near the electrode surface. Localisation of HRP and TMB to the FNA network in the presence of microRNA was achieved with a tagged DNA reporter sequence. Synthetic miR-141 and miR-21 detection down to 1 aM and 10 fM, respectively, was achievable with this amplification-free method. Quantitative detection of miR-141 was additionally observed in PCa cell lines. This work could multiplex up to 16 individual sensors for microRNA detection [96]. Further expansion of microRNA probes for this FNA platform could result in simultaneous high-throughput detection of multiple nucleic acid markers for PCa diagnosis and prognosis.

For example, a screen-printed carbon electrode (SPCE)-based biosensor developed by Chiou et al. was capable of detecting exosomal miR-451 and miR-21 in PCa urine samples [83]. Screen-printing electrodes are a disposable, cheap alternative to traditional methods, ideal for PoC purposes [98]. NeutrAvidin binding to a biotinylated ssDNA probe was utilised to tether the probe to the screen-printed carbon electrode (SPCE)-based biosensor [83]. A further probe complementary to part of the microRNA sequence was tagged with fluorescein. Upon binding of the target microRNA, HRP attached to an anti-fluorescein antibody was localised to the SPCE for chronoamperometry measurement (Figure 2b). Selectivity of the device for these exosomal microRNAs was confirmed against miR-141 and miR-636. With this biosensor, a threshold of 220 nA for exosomal miR-451 could distinguish between PCa patients and a non-cancer group (BPH patients and healthy people). Exosomal miR-21 detection in PCa samples correlated with tumour size in PCa but was not elevated in PCa relative to the controls [83]. As such, detection of these two microRNAs could provide both diagnostic and prognostic utility directly in the clinic.

These works illustrate the versatility of chronoamperometry as a bioelectrical technique for detection of multiple nucleic acid biomarkers for PCa diagnostics and prognostics.

### 3.3. Potentiometric Sensing Using Field-Effect Transistors

FETs when used as sensing elements for diagnostics typically have portable instrumentation, low power usage and cost-effective manufacturing processes, making them an exemplary electrochemical technique for PoC purposes. In particular, some FETs can be utilised with unmodified complementary metal-oxide semiconductor (CMOS) technology, simplifying the manufacturing process of these devices. Organic FETS (OFETs) are typically functionalised with biorecognition elements for nucleic acid sensing. For example, miR-1246, miR-21 and let7b were simultaneously multipanel-detected with a reduced graphene oxide nanosheet and immobilised peptide nucleic acid microRNA probes [21]. Partitioning of the device for individual microRNA detection allowed for simultaneous detection of the three microRNAs. The formation of PNA-microRNA hybrids near the FET surface resulted in an increased electrostatic charge that could be detected as a shift in the gate voltage (Figure 2c). Crucially, urine samples could be added to this microfluidic device without the requirement for pre-processing for quantitative microRNA detection down to 10 fM within 20 min. Elevated microRNA levels were detected in six PCa patients compared to four healthy controls, and these were ratified with reverse transcription-qPCR [21]. This work provided a strong foundation for a non-invasive microRNA detection system for direct and rapid PoC testing. Evaluation of the clinical validity of microRNA detection with this device and a larger cohort of PCa patients would further strengthen its implementation in clinics.

Direct detection of miR-21 from PCa blood serum samples was also observed with a solution-gated graphene FET designed by Deng et al. [20]. Induced Dirac voltage shifts from the binding of miR-21 to a complementary ssDNA probe on the gold gate surface can sensitively detect a relevant biomarker presence. An exemplary quantitative sensitivity of 0.01 aM, approaching single-molecule detection, was observed with this device. A single change of one nucleotide in the miR-21 sequence resulted in lower Dirac voltage shifts, confirming the specificity of the biosensor [20]. Direct testing in clinical blood serum samples of miR-21 could successfully distinguish between PCa patients and patients with benign prostatic hyperplasia (BPH). Since PSA testing is additionally elevated in other prostatic diseases, including BPH, circulating miR-21 PoC detection could provide additional diagnostic information for PCa patients.

Ion-sensitive field-effect transistors (ISFETs) are an FET that can detect the concentration of ions in solution through a passivation (sensing) layer. The passivation layer can detect changes in pH in the solution above through the hydroxylation of the layer detecting pertubations in ion concentration in the solution above [99]. ISFETs can be utilised directly with unmodified complementary metal-oxide semiconductor (CMOS) technology, increasing the ease of manufacture and implementation of ISFET PoC devices [100].

Previous utilisation of ISFETs with replacement of the traditional Si_3_N_4_ passivation layer with rare-earth oxide films has resulted in detection of AR-V7 cDNA for prediction of PCa drug resistance [74]. However, the need for qPCR pre-amplification of AR-V7 cDNA before detection of biomarker presence with an ssDNA probe currently limits this technique for a PoC setting. Adjustment to isothermal amplification techniques for pre-amplification could allow for direct PoC detection utilising this ISFET biosensor.

The authors have previously published RT-LAMP reactions coupled directly with ISFET biosensors and have previously been successful at detecting endogenous expression of PCa nucleic acid biomarkers with a handheld device named Lacewing [84,97,101]. Amplification reactions produce a proton for each nucleotide addition to a DNA strand [102]. When the target nucleic acid is present, the pH change from the amplification reaction can be detected with the ISFET biosensor (Figure 2c). This biosensor does not necessitate functionalisation of the ISFET surface, reducing the associated cost and complexity of the device. With this method, AR-V7, AR-FL, TMPRSS2-ERG and YAP1 mRNA were sensitively detected in real-time within 30 min with the Lacewing device. Down to 5 aM mRNA of each target (8 aM for AR-FL) was achievable with this technique, and specificity was confirmed with RNA extracted from PCa cell lines [84,97]. Synthetic detection in both serum and plasma additionally took place. Testing of this electrochemical device with clinical samples could result in valuable prognostic and predictive data for rapid clinical decision making.

## 4. REASSURED Criteria and Future Directions for PCa PoC Devices

The electrochemical devices explored in this review present simple, sensitive and specific methods with PoC compatibility for PCa diagnostics and prognostics (Table 1). However, establishment of an electrochemical device or procedure that fulfils the REASSURED criteria has not yet been developed for PCa.

Many of the electrochemical techniques utilised for PCa diagnostics and prognostics have been employed for microRNA, mRNA and lncRNA detection platforms. EIS devices alone have not yet been utilised for clinical sample testing. SWV, DPV, chronoamperometry and solution gated-graphene FET are all capable of detecting attomolar concentrations of microRNAs. These exemplary sensitivities could allow for direct testing in blood or urine samples from PCa patients. Attomolar sensitivities for mRNA detection have also been observed with ISFETs and chronoamperometry electrochemical devices [84,95]. The majority of electrochemical devices have also been checked for specificity, either through detection in cell lines or through synthetic nucleic acids. Several electrochemical devices for microRNA detection are capable of discerning between the desired target and a sequence with one base pair mismatch [20,23]. Given the amount of redundancy in microRNA sequences, this testing is valuable, to reduce the likelihood of false positives [103]. In order for the electrochemical PoC tests to be clinically viable they should have high sensitivities and be robustly ratified for specificity testing, ideally with synthetic and endogenous RNA.

Several studies within this review have detected multiple endogenous nucleic acids from clinical samples from blood, urine or their extracts. Many of these studies report an ascribed benefit to detecting relevant PCa microRNAs or mRNAs biomarkers, but typically within small cohorts [42,95]. Previous validation of non-PoC nucleic acid tests for PCa has required robust clinical studies before deployment. This necessitates multicentre validation clinical studies to explore the robustness of the PoC tests and extensive investigation of improved patient outcomes when provided with clinical information from the developed assays [5,13,104,105]. In addition, while all the devices in this review have PoC potential, testing within a clinical setting has not taken place. Translation of these devices directly into hospitals will be crucial to establishing the devices’ utility and position within the current clinical framework. Fulfilment of the user-friendly stipulation in the REASSURED criteria would ideally require devices to be tested with non-specialised personnel.

Multiplex testing has additionally been successful with electrochemical PoC testing for PCa diagnostics. Quantitation of up to four nucleic acid markers simultaneously has been achieved for PCa Poc testing [95]. Other electrochemical devices have additionally shown capacity for expansion of multiplex capability. For example, Wen et al. utilised a 16*X* sensor chip where creation of further ssDNA probes could expand upon the three microRNA targets utilised in the study [96]. To combat the inherent heterogeneity of PCa, FDA-approved molecular testing typically utilises large panels of nucleic acid markers. The GPS, Decipher Score and CCR detect the expression of 17, 22 and 46 genes, respectively [5,7,106]. Augmentation of developed PoC PCa devices to accommodate large arrays of nucleic acid markers for simultaneous detection will likely improve the diagnostic or prognostic capabilities of the device.

A singular microRNA-detecting PoC device for PCa diagnosis and prognosis allows for direct testing from urine samples with minimal processing [20]. The ease of processing of this device allows for easy implementation of the devices into clinics [20]. Despite this, many other papers have illustrated the utility of microRNA detection directly from serum, which can be easily extracted from whole blood by a clinical laboratory. Alternatively, several papers have reported PoC-compatible sample preparation techniques that allow for appropriate conversion of blood or urine into relevant media for analyte detection in PCa [41,95]. Especially, in instances where copy numbers of the nucleic acid target in the biofluid are low—for example, circulating mRNA in the blood—some degree of PoC sample preparation is likely to be required for relevant PoC detection strategies.

Within the work explored within this review, Koo et al., Deng et al. and Kim et al. currently present the most advanced PoC tests towards clinical implementation [20,21,95]. Koo et al. have developed a multiplex chronoamperometry device capable of PoC sample preparation and subsequent nucleic acid detection from both urine and blood. Corroboration of biomarker presence within multiple biofluids could result in a more robust diagnostic and risk-prediction device. Additionally, the utilisation of 30 PCa patient samples and five healthy donors presents a relatively large patient cohort compared to many of the other PoC electrochemical devices. The devices developed by Kim et al. and Deng et al. are both capable of microRNA testing directly from patient urine and blood, respectively. The work from Deng et al. presented the highest sensitivity for nucleic acid detection, with miR-21 observed down to 0.01 aM. Expansion of this device to detect multiple microRNAs simultaneously would greatly increase its value for PCa diagnostics. Kim et al., while exhibiting a lower sensitivity of 10 fM, were capable of multiplex detection of three microRNAs directly from urine samples. As such, this device is more likely to provide valuable clinical information for a larger range of PCa patients. Expansion of the patient cohort from the initial proof of concept study, however, will be required, to robustly confirm the validity of the device in aiding PCa diagnosis.

While ctDNA can provide both valuable diagnostic and prognostic information for PCa, current PoC electrochemical techniques have not detected this type of biomarker. However, ctDNA has been utilised for molecular diagnostics for PCa. Prediction of resistance to poly(ADP-ribose) polymerase (PARP) inhibitors with BRCA1/BRCA2 mutational status has resulted in FDA-approved tests for PCa from Myriad Genetics and FoundationOne [107]. A non-PoC electrochemical biosensor has also detected levels of DNA methylation in circulation, appropriately distinguishing between PCa patients and healthy men [108]. In other cancer types, PoC detection of ctDNA cell line and synthetic DNA with ISFETs and LAMP has previously been established [109,110]. Therefore, ctDNA presents potential as a relevant biomarker for detection in liquid biopsies, and future PoC tests could utilise this nucleic acid subtype for predictive purposes in PCa.

Current PoC electrochemical device platforms present exceptional potential for the future of PCa diagnostic and prognostic testing. Personalised medicine approaches for PCa through electrochemical testing could result in rapid, cheap and easy-to-use biosensors capable of providing clinically relevant information at the PoC.

## Data Availability

No new data were created or analyzed in this study. Data sharing is not applicable to this article.

## Abbreviations

The following abbreviations are used in this manuscript:

PoC	point of care
PCa	prostate cancer
PSA	prostate-specific antigen
ADT	androgen-deprivation therapies
AR	androgen receptor
PCA3	prostate cancer antigen 3
ssDNA	single-stranded DNA
EIS	electrochemical impedance spectroscopy
AuNP	gold nanoparticle
SPCE	screen-printed carbon electrode
MWCNT	multi-walled carbon nanotubes
CV	cyclic voltammetry
SWV	square-wave voltammetry
DPV	differential-pulse voltammetry
FET	field-effect transistors
ISFET	ion-sensitive FET
PTEN	phosphatase and tensin homolog
AKT	protein kinase B
mTOR	mammalian target of rapamycin
